# Authenticity made visible in medical students’ experiences of feeling like a doctor

**DOI:** 10.5116/ijme.5cf7.d60c

**Published:** 2019-06-14

**Authors:** Angelica Fredholm, Katri Manninen, Hans Hjelmqvist, Charlotte Silén

**Affiliations:** 1 Department of Learning, Informatics, Management and Ethics (LIME), Karolinska Institutet, Sweden; 2 Karolinska University Hospital, Department of Infectious Diseases, Sweden; 3 School of Medical Sciences, Örebro University, Sweden; 4 Department of Learning, Informatics, Management and Ethics (LIME), Karolinska Institutet, Sweden

**Keywords:** Medical education, authenticity, autonomy, professional development, professional identity, communities of practice

## Abstract

**Objectives:**

To interpret the phenomenon of authenticity made visible in medical students’ experiences of feeling like a doctor, i.e., how authenticity took shape in narratives about feeling like a doctor in clinical situations where students were challenged to be independent and to a high degree make choices and clinical decisions.

**Methods:**

The conducted research was positioned within phenomenological hermeneutic research tradition, interpreting participants’ experiences in a life-world perspective using narrative inquiry. Fifteen medical students in their fifth year were interviewed focusing on clinical situations. An abductive analysis approach was used to discover patterns and to interpret data following a phenomenological hermeneutic research method for textual interpretation.

**Results:**

The analysis resulted in a thematic structure of findings: Opportunity to experience authenticity through creating relationships; Opportunity to experience authenticity through responsibility; Opportunity to experience authenticity through independence, managing wholeness, and follow-up processes; Opportunity to experience authenticity through being able to reason and discern. Overarching the four themes was the perceived need for attachment, i.e. attachment to patients, to supervisors, to the workplace, to the situation and reasoning and knowledge.

**Conclusions:**

Essential for the experience of feeling like a doctor was authentic situations that resulted in the experienced members of a community of practice and the perceived development of a professional identity. These findings can advance the understanding of how clinical education should be organized to facilitate professional identity development.

## Introduction

Studies in education show a connection between authenticity, autonomy in learning, and professional development. For the planning and execution of clinical education for medical students, it is important to understand medical students´ professional development and what makes them start to take on an identity as a doctor.  An understanding of how authenticity impacts on students’ learning and professional development is important for the future development of learning and teaching strategies in medical education.  However, there is an existing knowledge gap concerning authenticity as a phenomenon featured in a medical education context, and therefore a gap in what we know about the development of clinical education that facilitates and supports experiences of authenticity and consequently professional identity development.

Experiencing meaning and relevance is seen as vital for learning.^1^ Studies in a clinical setting showed authenticity as an indirect driving force for learning by creating meaning and relevance.^2^^-^^4^ In these studies, meaning, and relevance appeared when students’ actions and decisions had a real, actual impact on, and consequence for, patients’ care and wellbeing. Findings also showed that reality itself was not enough but meaning occurred when students’ actions and decisions had an actual impact on the patient, when they mattered and when students were allowed to form a relationship and feel a moral responsibility for the patient. Analyzed from a social perspective based on the Social Theory of Learning ^5^^,^^6^ findings from an earlier study 2 also suggest that authentic clinical situations enhanced students’ experiences of autonomy. Autonomy is in the literature described as an important part of identity^7^^,^^8^   and thus, we suggest that authenticity might play a role in the development of professional identity. Hence, there is an existing knowledge gap concerning authenticity as a phenomenon in depth featured in a medical education context, and therefore a gap in what we know about the development of clinical education that facilitates and supports experiences of authenticity. For these reasons, we need to know more about authenticity as a phenomenon.

Authentic learning experiences are considered to play a role in the transition from student to professional practitioner.^9^ Four different theoretical frameworks can be identified in the literature regarding the nature and definitions of authentic learning.^9^^,^^10^ These frameworks are Real-world experiences that match the characteristics of professional activity within a certain community; The sociocultural perspective offered by Lave and Wenger where students are allowed to begin to interact with some of the routines, rituals and conventions of a profession within a Community of Practice (CoP); Authentic learning defined by the student’s perception of the authenticity by the task at hand, and The context in which the learning occurs and the nature of the learning experience that must assist students to make connections between new experiences and their current understanding. Authenticity in a philosophical sense is about those things that genuinely define people as who they are, and what being human means to them. Authenticity can be defined as socially situated,^12^ involving knowing who we are in our social world, understanding how that world shapes us and how we position ourselves in that world. Authenticity is multifaceted and is comprised of at least four parts: “being genuine, showing consistency between values and actions, relating to others in such a way as to encourage their authenticity, and living a critical life”.^13^ Being critical in this sense is about critical reflection upon oneself and others, and becoming authentic through a transformative learning process.^12^ Authentic learning can be defined as a dynamic relationship between the development of personality and cultural practices.^14^  Connections are made between educational authenticity and what it means to live authentically, and by doing so brings to the fore that which lies at the core of education is “not learning, truth and knowledge, but thinking, meaning and understanding”.^15^ This heart of education is found in relationships – in a dialogical relationship between oneself and others rather than in bodies of knowledge. When viewed about curriculum development, what makes a curriculum, problem or assessment authentic, is not merely a correspondence to the likeness of what is in the world, but also to what it ought to be - thereby introducing a moral or a value side to authenticity.^15^ A literature review performed to gain insight into the meaning of authenticity in teaching in adult and higher education settings, reveals authenticity as a complex and multidimensional phenomenon.^16^ Authenticity in teaching involved features such as “being genuine, becoming more self-aware, being defined by oneself rather than by others’ expectations, bringing parts of oneself into interactions with students, and critically reflecting on oneself, others, relationships and context, and so forth”.^16^ It is suggested that for authenticity to be meaningful and not just lie in oneself, it needs to be sought in relation to issues that matter crucially, and for education, this means issues of genuine interest and significance to the learner.^16^ Furthermore, it was proposed that the genuine dialogue found in authentic relationships^17^ translated to higher education, is a dialogue that centers on ideas that really matter to students. Authenticity, or self-authorship, is claimed to be an important outcome of higher education and a prerequisite for students to cope with uncertainty and complexity.^18^ It has been showed how authenticity in clinical education was two-dimensional with external authenticity created by the educational system and the surrounding environment and internal authenticity as an experience within the student.^3^^,^^4^ External authenticity referred to the actual reality, such as meeting real patients and being in a real clinical setting, whereas internal authenticity is achieved by students creating mutual relationships with patients and having a feeling of belonging. Internal authenticity can further be described as students’ feelings of contributing and being a valid member of the team.^19^

Self-determination theory distinguishes between autonomy and independence.^20^ Autonomy does not mean working on one’s own but working out of one’s own choice with no feelings of pressure. Translated to the care of patients, this would mean giving students opportunities to handle patients on their own and to feel that they have support from supervisors.^21^ Autonomy with structure is important for self-regulated learning^22^ and autonomous motivation is associated with an optimal challenge; creating flow when the degree of challenge is right^23^ and feelings of being controlled when the pressure becomes too high. In line with this reasoning, literature^24^ claims that behaving autonomously does not mean behaving independently, but rather behaving of one’s own volition and in accordance with one’s inner self. Thus, supporting autonomy in learning means not to be distant, vague or leaving students to manage on their own, but rather to set standards, create limits and give feedback in a way that provides choice, acknowledges feelings and lets students find solutions and answers on their own. There are arguments for the need to broaden the view of autonomy from simple questions of control, to questions about constructing personal identity, not only through the learning process but also through the learner's interpersonal relationships.^7^ In this view, autonomy is the prerequisite for the development of individual identity,^7^ and identity^8^ is “the subject's inclusion in a structured relationship of interactions”. This means that autonomy is something that takes place “through reciprocity based on exchange and otherness” and “supposes that autonomy is born out of a realization of the interdependence of people who are summoned and bound to construct a social contract”.^8^ The process of becoming a professional is open-ended and incomplete. This process entails developing and refining an embodied understanding of the professional practice, thus integrating knowing, acting, and being in the world in the form of professional ways of being. This process does not entail fixed stages but is individual and unfold as a transition over time.^25^ Lave and Wenger^5^ claim that the development of identity is central to the careers of newcomers in communities of practice, and thus central to legitimate peripheral participation. Legitimate peripheral participation as described by Lave and Wenger as a way for students to take an active part in practice and to experience the physical environment, the affordances, activities, language, artifacts and so on within a community of practice without demands on full participation. Newcomers need to have broad access to areas of a mature practice, but at the same time, productive peripheral participation requires less demands on time, effort and responsibility than for full participants. Legitimate peripheral participation is more than a process of learning, but a reciprocal relation between person and practice.

The move towards full participation does not take place in a static context, and changes over time and circumstances.^5^ Through a systematic review of the literature on professional identity in higher education, it was found that most studies identified a strong link between reconciling personal values with professional when it came to the values, morals, and dispositions that underpinned future professional practice.^26^ The key message from the reviewed literature was that professional identity development was about being in the world constituted by various settings and communities, thus making professional identity development complex and dynamic. Furthermore, the literature review identified the overall agreement that professional identity development is facilitated by collaborative and dialogic learning from practice and that real experiences foster it in practice. Professional identity development is fluid and implies a shift and transformation of personal and professional knowledge, skills, and dispositions.^26^ Thus, to capture experiences of authenticity, there was, therefore, a need to identify students in the process of forming a new professional identity. This study aimed to interpret the phenomenon of authenticity made visible in medical students’ experiences of feeling like a doctor, i.e., how authenticity took shape in narratives about feeling like a doctor in clinical situations where students were challenged to be independent and to a high degree make choices and clinical decisions.

## Methods

The research conducted is positioned within the phenomenological hermeneutic research tradition describing and understanding phenomena, and their meaning revealed through interpretation. As such, the focus lies on understanding phenomena as represented in peoples’ life-world and the interpretation of the meaning of these phenomena. ^27^^-^^30^ It’s argued here that the life-world is mediated through narratives where individuals' subjective understanding and sense-making of their life-world become visible.^27^^-^^30^ The meaning of a phenomenon in a person’s life-world becomes visible through narratives concerning experiences of this particular phenomenon. Narrative inquiry was used and unstructured interviews with two open questions conducted. Interviews were interpreted using phenomenological hermeneutic analysis with the theoretical analysis. The use of theory in this study to interpret, deepen, and illuminate findings is part of abductive research design. In an abductive analysis of data, the theory is used before or together with the empirical data. Using abductive reasoning during the research process, there is consequently an alternation between theory and empirical data, where both are interpreted in the light of each other. Abduction is to, with the use of existing knowledge, find theoretical patterns or “deep structures”, which explain or help understand the empirical inductive patterns or “surface structures” that are observed and interpreted.^31^

### Context and participants

A convenience sample was used, and a group of participants with the first-hand experience with the phenomenon under study was chosen. Informants were medical students in their last semester experiencing clinical practice in a primary healthcare setting with ample possibility for independent patient contact. The medical program in Sweden is a five-and-a-half-year undergraduate program, resulting in a master’s degree in medicine. Fifteen interviews were conducted with medical students finishing their last (11^th^) semester at a problem-based medical program, towards the end of a seven-week clinical placement in primary health-care (for two participants after finishing that placement).  All but two primary health-care providers (affecting three students) were active in an educational development project called “academic primary health-care”, supporting learning for students, staff, and patients and strengthening relationships between health-care and the university. All primary health-care providers were located in small rural towns. Nine students were female, and six were male, between the ages of 24 and 38 with a mean age of 28 years. Almost every student had parents with an academic background. Six students were married or living together with a partner. All students had experience of work in the health-care sector, and two students had a former career within the health-care sector. All students had held temporary positions as junior doctors or medical assistants at later stages in their education. Students were invited to participate in the study via a written letter on the internal course website. This, however, did not generate any response, so an information meeting was set up and gave the researcher the possibility to inform in person about the purpose and implications of the study. At the end of the meeting, students interested in participating filled out a contact form allowing the researcher to contact them further and decide on time and place for interviews.

The ethical principles adopted in this study that adhered to the principles of anonymity were possible to secure data processing, transparency and minimality, and lawful data collection. Ethical approval was obtained from the Regional Ethical Review Board in Stockholm. All participants received an information letter with a description of the aim and context of the study. The letter stressed voluntarily to participate and pointed out the right to confidentiality. Each interview opened with a presentation of the study and participants signing the informed consent paper. Once again, confidentiality and the right to withdraw participation were stressed. The interviewer had no previous knowledge about or connections to the participants previous to or after the study to minimize the risk of any dependency issues.

### Data collection

The study was undertaken using narrative inquiry. Stories were collected as a means of understanding experiences as lived and told, through both research and literature.^30^ The term narrative is used here to explain human experience and human meaning-making of materials and circumstances.^32^ Interviews took place at the clinical work in the student’s office or a conference room and lasted between approximately 40 and 80 minutes. Narratives were captured by asking two open questions; “Tell me about a situation or situations when you feel like a doctor” and “Tell me about a situation or situations when you do not feel like a doctor”. These questions were chosen to gain rich narratives and ensure a wide scope of inquiry. To be able to secure as rich narratives as possible, a wide range of follow-up questions were asked, such as “tell me more, why is that do you think, can you describe more in detail, why do you think it was like that?” etc. To check the interviewer’s understanding, recapitulation and summaries were used during the interviews. Each interview started with a set of more “common” questions and issues regarding background variables, slowly moving towards deeper layers of meaning about the phenomenon under inquiry.  A log book was kept capturing the researcher’s reflections and thoughts about each interview. After fifteen interviews, the decision was made that there was sufficient data to interpret the phenomenon under study in depth, due to the rich material and thick descriptions. A person made a verbatim transcription of interviews outside of the research team who had experience in transcribing research material. Longer pauses and evident expressions of emotions were noted in the transcripts.

### Data analysis

Drawing on the work of Ricoeur and his work on textual interpretation as a movement from understanding to explanation, and from explanation to comprehension. The data analysis consisted of three parts, following a well-established phenomenological hermeneutic research method for textual interpretation of interviews.^29^ This method consists of Naïve reading, Structural analysis, and Comprehensive understanding/Interpreted whole. The naïve reading was conducted to grasp the whole meaning of the text and to create an openness to the text. The naïve reading enables the researcher to switch from a natural understanding to a more phenomenological attitude and guide the consequent structural analysis.^29^ The structural analysis aims at creating a movement from understanding to explanation, thus in the words of Ricoeur,^27^ moving from what the text says towards what it talks about. The purpose of the structural analysis is to create distance to the text, enabling the researcher to view the text differently.^29^ In this study, a structural analysis was undertaken where condensed narrative elements or meaning units were extracted and sorted together with keywords/concepts emerging from the naïve reading. The combined meaning units were read again and checked against the original text. The thematic structure here provides the hierarchy of themes and the themes the content and meaning. The themes and the overarching theme were reflected on in relation to the naïve understanding to validate or invalidate that first interpretation.^29^ The comprehensive understanding or the interpreted whole is arrived at by reflecting on the themes and main themes in relation to the research question and the investigated phenomenon.^29^ The preunderstanding of the researchers was analyzed in relation to the findings. Using literature that helped revise, widen and deepen understanding, the overarching theme “Need for attachment,” was analyzed and illuminated through a model for transformative learning and authenticity in a clinical education ward^34^ and the theoretical framework of Communities of Practice. The interplay between art and science in conducting a phenomenological hermeneutic study exists between the artistic element that is the formulation of the naïve understanding, the scientific talents used in the structural analysis and the critical talent to arrive at and formulate a comprehensive understanding.^29^ In this study, this was viewed as the interplay between the researcher’s ability to understand and relate to the students and their lived experience, and the ability to use and apply the relevant theory. Here data analyses were carried out with great care, with more than one interpreter reaching consensus, and with adherence to the method described. To obtain as rich and thick descriptions as possible, narrative interviews were conducted with one large open question about the investigated phenomenon. As the interviewees only can understand and narrate their lived understanding in relation to their preunderstanding, and the interviewer understand and interpret in the same way, there is a risk for misunderstanding.^29^ However, during the interviews in this study the risk was minimized by asking follow-up questions like “how do you mean”, “can you explain more” or “elaborate”. The number of follow-up questions and validating questions asked during the interviews also ensured a depth in the interviews. Characteristic of the interview process was a good climate and a good relationship with the informants. Between the formulation of the naïve understanding and the structural analysis of findings a considerable time of half a year had elapsed, contributing to the desired distance to the text, mainly as a result of the structural analysis. The distance also became visible through the change from the more tangible naïve understanding of the abstract formulation of themes. In the comprehensive understanding, where findings were analyzed and interpreted through existing theory, findings became transferrable to other contexts. The researcher's preunderstanding is vital to be able to conduct this step of the analysis, and this step also helps manage that same preunderstanding and minimize the risk of over-interpretation of findings. Two other researchers took part in the analysis, and each step throughout the analysis was carefully discussed within the research team. In the presentation of findings, quotes are used to enhance credibility. The use of theory in the interpretation of data in this study allows for a high degree of transferability and reflexivity, which widens the possible contexts to which findings can be applied.

## Results

Results consist of Naïve reading, Structural analysis, and Comprehensive understanding combined and should be understood as a whole, but each part is here presented separately. Themes belonging to the structural analysis are illustrated with longer quotations chosen to capture the essential meaning of each theme.

The transcribed interviews were read as a whole several times, creating a naïve understanding of the text that led us to understand the experience of “feeling like a doctor” as consistent with having to do with a sense of belonging, a need to manage on one’s own, ability for clinical reasoning, a need to be able to create relationship with patients and supervisors and  the importance of theoretical knowledge. In our naïve understanding of the narratives, students felt like doctors when they were able to act on their own and were allowed to form relationships with patients and matter to patients. Clinical reasoning was highlighted as relevant in the experience of feeling like a doctor, and this entailed being able to ask the right questions and to discern relevant parameters and symptoms. In connection with this clinical reasoning, the relationship with the supervisor was stressed as important for feedback. Theoretical knowledge was perceived as important to be able to function as a doctor.

The structural analysis resulted in a more abstract and interpreted thematic structure of findings with four themes and one overarching theme. The themes were: Opportunity to experience authenticity through creating relationships; Opportunity to experience authenticity through responsibility; Opportunity to experience authenticity through independence, managing wholeness, and follow-up processes; Opportunity to experience authenticity through being able to reason and discern. Overarching the four themes was the perceived need for attachment, i.e. attachment to patients, to supervisors, to the workplace, to the situation and reasoning and knowledge and the meaning created from this attachment. The detachment was perceived when supervisors took over or interfered with processes or relationships created by the student. Larger sets of quotations here exemplify the thematic structure.

### Opportunity to experience authenticity through creating relationships

Findings show the importance of creating relationships. The nature of the relationship with the clinical supervisor is at the forefront of the clinical placement, together with the relationships formed with the patient. When allowed to form these relationships, students could, even for a shorter period, feel like the patient’s doctor or like a colleague to the supervisor. Also, relationships with other professions at the clinical placement mattered, and to be recognized by other professional groups made students feel like doctors. Contributing to the sense of being a doctor was the feeling of belonging, and that you as a student, played a real part in the clinical work, that you filled a role and was useful. This was especially evident when students felt that they had succeeded in helping the patient and that the patient trusted them and was pleased with the care given and the actions taken as a result of the consultation.

One student described the feeling of being included and seen by other professional groups:

“…there are quite a lot of other professional groups…such as diabetes nurses…I have felt that they also give me feedback. If they have a patient, then they tap me on my back and ask if I can check this patient. They don’t do that. Otherwise, they go to one of the resident doctors…but they have come to me and asked me! If there is a patient with diabetes that I have met earlier, and that now three weeks later is here with some changes in laboratory results, than she (the nurse) will write to me and say; you met with this patient, and now it looks like this…what do you think…?... They also send word to the clinical supervisor, but to me as well, I am included…even other professional groups are interested in what I do here, and this has made me feel more included, a bit like I am a doctor…” (Student No 5, male, age 25, limited professional experience)

A learning relationship with the clinical supervisor was characterized by the supervisor as a source of feedback, security, the opportunity for discussion, and someone to test your thoughts and knowledge with. When at its best, the relationship was described like that to a senior colleague. The need for security and control provided by the supervisor differed between students, but also between situations. Sometimes, the supervisor was needed as “a buffer” between the student and the reality and as a source of safety and assurance. One of the students described how the relationship to the supervisor impacted on the relationship with her patients:

“…If I feel that if the relationship to the supervisor isn’t working, then it becomes much harder to feel confident and actually make your own decisions… …and go further together with the patient. If I feel safe that we think alike.  If I know how my supervisor will think and assess…then I can at least in advance say to my patient that “we will probably handle it like this”…but if I feel insecure whether or not the supervisor will do something else, then I can’t give such feed-back to the patient….I don´t feel safe to say anything to the patient, I will back off a bit and let the supervisor take that part instead.” (Student No 11, female, age 30, background in another caring profession)

### Opportunity to experience authenticity through responsibility and independence

Students described how they felt like doctors when they had the opportunity to interact alone with patients and how they automatically took on more responsibility when they could handle a patient case by themselves. Even if the clinical supervisor “visited” the patient consultation, it was important for the students to complete the consultation alone with the patient. This completion, together with the patient made students feel like doctors. Important features of handling a patient case by themselves were that students themselves decided on the course of action and made choices before checking with the clinical supervisor. If the supervisor then concurred with the student’s assessment, a strong feeling of being a doctor emerged. Connected to the feeling of independence was also the purely practical aspect of having one’s own space, e.g., office or consultation room. Another important factor in feeling like a doctor was being able to perform practical tasks without support from the supervisor. Sometimes the experience of feeling like a doctor was taken away from students and the supervisor was perceived as an interference in the ongoing relationship between the student and his or her patient. One of the students reported how she became more thorough if she knew that her actions really mattered:

“…If you know that every patient will be examined again by someone else, you don’t become as…maybe… thorough, or…you don´t have to trust what you do yourself as much. So you reason more if you don’t ask someone to come and check afterward…you assess everything more carefully and separately…I don’t think that I think it through as thoroughly if I know that someone else is coming in after me to reexamine the patient…"  (Student No 2, female, age 36, multiple professional experiences)

### Opportunity to experience authenticity through managing wholeness and follow up processes

The clinical placement in primary health-care offered students a possibility to manage wholeness in the sense that they were given the opportunity to observe patients’ problems in a context and follow the patient from initial consultation through treatment to the evaluation of the actions taken. Students described the impact of being able to follow up patients and patients’ situations and problems. This created a dimension of learning with feedback on actions taken and decisions made that students experienced as contributing to the feeling of being a doctor. Follow-up of processes initiated by the students themselves also created a feeling of having “their own” patients and really being the doctor for these patients. One student described the importance of being able to follow-up processes for his learning:

“I have said that this is among the best here, that you, in some way, through this signing and authentication… because you can enter (the patient’s medical record) and see what happened if the patient has seen someone else and see ok; this is how it all went (…) I think this is really important…for learning…you must be able to follow up and see what happened with the patient. I think this is the most important for my personal development and for me to become a better doctor, otherwise, I don´t know what I have done right or wrong…” (Student No 13, male, age 30, background in another caring profession)

### Opportunity to experience authenticity trough being able to reason and discern

To a great extent, the experience of feeling like a doctor had to do with the ability to reason. Students highlighted the importance of determining which questions to ask, and to discern relevance among variables. Reasoning also had to do with prioritizing. Being systematic and experience that you can connect clinical findings to theory brought forth the feeling of being a doctor. Much emphasis was placed on these more cognitive skills. Also, tolerance of uncertainty was connected with feeling like a doctor, i.e. being able to handle the fact that you don´t know but use your judgment and still decide on a course of action that you think is best in the present circumstances.  Some students also stressed that they felt like doctors when they understood facts and theory. One of the students describe the importance of developing clinical reasoning skills like this:

“…everyone can hear a heart murmur, but to connect the heart murmur to symptoms and try to reason, this is where I feel that the whole theoretical education one has had, this is where one is supposed to connect isolated parts.., this is where the puzzle is put together…when I was in semester three, I could not in any way reason. I could in the same manner as now, ask relevant questions, maybe not entirely, but tolerably relevant questions…but the further you get, the more you have seen and learned, and the more you can connect clinical findings to…well, medical conditions really…” (No 10, male, age 27, caring work experience)

### Comprehensive understanding

In order to further decontextualize findings and to extend the analysis and create a new comprehensive understanding, we first applied a model for transformative learning and authenticity in a clinical education ward.^34^ This model illustrates student learning at a clinical education ward as a transformative process where experiences of both external and internal authenticity form the basis for creating learning relationships, resulting in students’ learning and understanding of the professional role. This experience of authenticity and learning relationships is potentially troublesome and described concerning a “liminal space” with “thresholds”,^34^ i.e. as a period of uncertainty for the student where the support of the supervisor becomes pivotal for crossing the threshold of understanding the professional role.  This model enabled us to interpret the themes by using a theoretical lens, and thus, authenticity was seen as a prerequisite for being able to form learning relationships with patients, supervisors, and other professionals. Through these authentic experiences and learning relationships, students were able to understand (and perceive) the professional role. In other words; findings showed that essential for the transformative learning experience of feeling like a doctor, were authentic internal experiences enabling learning relationships.

The overarching theme “Need for attachment”, seen through the lens of the model for transformative learning and authenticity in a clinical education ward,^34^ was in the comprehensive understanding interpreted as a need to belong, and the necessity of this sense of belonging for experiencing the professional role became evident. This deepened perspective allowed us to widen the analysis further by incorporating social theories for learning in relation to our findings. Thus, findings were interpreted by attaching the theoretical framework of Communities of practice 5, and in doing so the comprehensive understanding grew and gained more depth. This interpretation of findings gave that the experience of feeling like a doctor is equal to perceived membership of the CoP of the primary health-care placement and more specifically that of the doctors there. In the Theory of communities of practice^5^^,^^6^ learning is defined as consisting of the components belonging, becoming, experience, and doing. These components are interconnected and mutually defining, meaning that either of the components can be placed in the primary focus. Deriving from this reasoning, we can define belonging as consisting of becoming, experiencing, doing and learning, i.e. membership in a community of practice is distinguished by being able to take part, learn and experience in that practice, thus changing identity. Our findings show how membership was achieved by being able and allowed to take part in the practice of CoP and to be able to create meaning out of that practice. In doing so, a change in identity emerged, here seen as professional becoming i.e. feeling like a doctor. A threat to this perceived membership and the experience of feeling like a doctor was the interference or sometimes even the presence of the supervisor. A prerequisite for being able to take part in the practice of the community was authentic internal experiences. In short; authenticity was essential for creating learning relationships enabling transformative learning processes that resulted in the experienced members of the CoP of the primary health-care placement and the doctors there.

## Discussion

In order to transfer findings, we here discuss them against sociocultural perspectives on learning and professional identity development. The development of a professional identity is a complex process and although many factors are contributing to the development of a professional identity such as a persons’ personal identity, the key message from a literature review of higher education literature regarding professional identity,^26^ predominantly concerned the dynamic, transformative nature of professional. The collective and dialogic nature of learning from practice was highlighted, but with a strong emphasis on the individual learner. The transformative and collective nature of learning is often addressed with sociocultural perspectives on professional identity development, foremost represented in the theories regarding the Community of practice. Factors contributing to learning are such as the opportunity for reflection, meaning making, critical reasoning, and authentic learning experiences.^26^

The social theory of learning^5^ distinguishes three modes of belonging to a community connected to the formation of identity; imagination, alignment, and engagement. Imagination has to do with a person’s ability to create images of the world and seeing connections through time and space concerning our own experience. Engagement is about an ongoing negotiation of meaning through relationships, interactions, practices, and shared stories of learning. Alignment is, achieved by shared discourses, coordinated enterprises, styles, complexity, and compliance.^5^ Engagement and alignment becomes especially fruitful in analyzing our findings in the view of perceived threats to membership of the CoP, in findings expressed as the clinical supervisor “taking over” and diminishing the experience of feeling like a doctor in relation to the patient, to the caring process, to student independence and to the student’s clinical reasoning. “Viewed as an experience of identity, learning entails both a process and a place. It entails a process of transforming knowledge as well as a context in which to define an identity of participation”.^5^ As a result of this, “to support learning is not only to support the acquisition of knowledge but also to offer a place where new ways of knowing can be realized”.^5^ Such a place is described as “legitimate, peripheral participation” where students can participate in a CoP and partly participate in the ongoing professional action in this CoP.^11^ As it would seem in our findings, perceived membership varied from experience to experience, thus making it a seemingly “unstable entity” for these students on a gliding scale. Possibly, there is an argument for that in the case of the clinical supervisor stepping in and taking over, the acquisition of knowledge might still be intact, but the place to realize this knowledge – the doctor-patient relationship – is interfered with or failing.

As described, authenticity has several dimensions; being human and living an authentic life true to your being, responding to and authentically caring for others, being authentic as a teacher and authentic learning encompassing identity. This study focuses on authenticity as experienced in learning, whereas the other dimensions mentioned above feature authenticity as a form of being. The link between these dimensions can be found in the development of identity – or professional identity – as this is a combination of being someone in the world and becoming that someone through authentic experiences. Overarching themes in findings were the perceived need for attachment (attachment to patients, to supervisors, to the workplace, to the situation and to reasoning and knowledge) and the meaning created from this attachment. The strong emphasis on relationships in findings and the formation of “real” relationships with patients gave students a sense of meaning. This can be interpreted as creating authentic encounters, responding to, and caring for, others in an authentic way, appreciating their inherent authenticity. Through the theoretical analysis of findings, we interpret the experience of feeling like a doctor and being a part of a CoP with authenticity as a prerequisite for this membership. Membership and participation in a CoP and the construction of identity are intimately connected, both in our findings as well as in the medical education literature.  Students’ need to participate and contribute to the practice of the CoP might be seen as a need to incorporate one’s whole person in this practice, to be seen as a valid member and be acknowledged as a whole person – and not just as a student.

Corresponding to the development of the concepts of internal and external authenticity,^34^ authentic learning activities were described as similar to “real” practice conducted within a culture.^10^ Here, authentic learning is explored by comparing it to legitimate, peripheral participation within a real community of practice^11^ where the environment and learning opportunity are authentic, but without the expectation of full participation. The difference, as we see it, between the peripheral participation of a CoP^11^ and the experienced membership seen in this study, is the lack of internal authenticity.^34^ The internal authenticity that played a key part in forming the necessary relationships that transformed identity could not be achieved through merely the space for participation and learning created in the CoP, the peripheral participation, the external authenticity. Thus, it could be argued that internal authenticity plays a key part in the development of a professional identity.

### Limitations

This study has some limitations. Even though the rich material and thick descriptions generated generous data material, the study could have benefited from a larger perspective, such as medical students from other universities. Another limitation is that the interviews are done by one researcher only, which could have an impact on the interpretation.

## Conclusions

Essential for the experience of feeling like a doctor was authentic internal situations that resulted in the experienced membership of a community of practice and perceived development of professional identity. These findings develop the understanding of how clinical education should be organized, and thereby provide an opportunity for students to form relationships with patients, future colleagues, other professional categories, and with each other as learners. There are implications for clinical education, such as the level of student engagement in the clinical workplace, the length of clinical placements, and how to make use of the student’s knowledge. Placements need to provide the opportunity to form authentic relationships with patients, supervisors, the workplace, and other professional groups. Future research needs to in-depth investigate the connections between authenticity and professional identity development.

### Conflict of Interest

The authors declare that they have no conflict of interest.
